# Efficacy and Cost-effectiveness of Promotion Methods to Recruit Participants to an Online Screening Registry for Alzheimer Disease Prevention Trials: Observational Study

**DOI:** 10.2196/26284

**Published:** 2021-07-22

**Authors:** Kenichiro Sato, Yoshiki Niimi, Ryoko Ihara, Kazushi Suzuki, Tatsushi Toda, Atsushi Iwata, Takeshi Iwatsubo

**Affiliations:** 1 Department of Neurology University of Tokyo Hospital Tokyo Japan; 2 Department of Neuropathology Graduate School of Medicine The University of Tokyo Tokyo Japan; 3 Unit for Early and Exploratory Clinical Development The University of Tokyo Hospital Tokyo Japan; 4 Department of Neurology Tokyo Metropolitan Geriatric Medical Center Hospital Tokyo Japan; 5 Division of Neurology, Internal Medicine National Defense Medical College Saitama Japan

**Keywords:** online clinical study, promotion, advertisement, cost-effectiveness, Trial-Ready Cohort, preclinical Alzheimer disease, clinical trial, Alzheimer, dementia, recruitment

## Abstract

**Background:**

Web-based screening may be suitable for identifying individuals with presymptomatic latent diseases for recruitment to clinical studies, as such people do not often visit hospitals in the presymptomatic stage. The promotion of such online screening studies is critical to their success, although it remains uncertain how the effectiveness of such promotion can differ, depending on the different promotion methods, domains of interest, or countries of implementation.

**Objective:**

The Japanese Trial-Ready Cohort (J-TRC) web study is our ongoing online screening registry to identify individuals with presymptomatic Alzheimer disease (AD), aimed at facilitating the clinical trials for AD prevention. Within the first 9 months of its 2019 launch, the J-TRC web study recruited thousands of online participants via multiple methods of promotion, including press releases, newspaper advertisements, web advertisements, or direct email invitations. Here, we aimed to quantitatively evaluate efficacy and cost-effectiveness of each of these multimodal promotion methods.

**Methods:**

We applied the vector-autoregression model to assess the degree of contribution of each type of promotion to the following target metrics: number of daily visitors to the J-TRC website, number of daily registrants to the J-TRC web study, daily rate of registration among visitors, daily rate of eligible participants among registrants, and median age of daily registrants. The average cost-effectiveness for each promotion method was also calculated using the total cost and the coefficients in the vector-autoregression model.

**Results:**

During the first 9 months of the reviewed period from October 31, 2019 to June 17, 2020, there were 48,334 website visitors and 4429 registrations (9.16% of 48,334 visitors), of which 3081 (69.56%) were eligible registrations. Initial press release reports and newspaper advertisements had a marked effect on increasing the number of daily visitors and daily registrants. Web advertisements significantly contributed to the increase in daily visitors (*P*<.001) but not to the daily registrants, and it also lowered the rate of registrations and the median age of daily registrants. Website visitors from the direct email invitation sent to other cognitive registries seem to have registered with the highest reliability. The calculated average cost-effectiveness for the initial press release was US $24.60 per visitor and US $96.10 per registrant, while the calculated average cost-effectiveness for the newspaper advertisements was US $28.60 per visitor and US $227.90 per registrant.

**Conclusions:**

Our multivariate time-series analysis showed that each promotion method had different features in their effect of recruiting participants to the J-TRC web study. Under the advertisement condition settings thus far, newspaper advertisements and initial press releases were the most effective promotion methods, with fair cost-effectiveness that was equivalent to earlier online studies. These results can provide important suggestions for future promotions for the recruitment of presymptomatic participants to AD clinical trials in Japan.

## Introduction

Web-based clinical studies are rapidly growing in importance to become one of the promising methods of clinical research [1], especially, in the era of COVID-19 [2]. In addition to targeting already developed diseases, a web-based approach such as an online survey may also be particularly suitable to target presymptomatic, latent diseases [3-5] or for presymptomatic disease screening [6], because asymptomatic individuals cannot always be expected to visit hospitals of their own volition.

Study advertising to reach the target population is critical to the success of web-based clinical studies [1,7]. Currently, there are several types of methods available to promote clinical studies [3-6,8-13], such as advertisements in standard media like newspapers [14] or television [8], online advertisements (eg, Google, Yahoo, or Facebook) [1], social media posts (eg, Facebook, Twitter, or Instagram) [8], direct phone calls or emails [8,15], or word of mouth [9]. Which of these modalities may be more effective for the target population might vary depending on the disease domain of interest, design of the clinical study, or regional environments associated with the clinical studies [5]. For example, it is reported that Japanese people in their 60s regard the newspaper as the more reliable news media than the internet or television [16], which suggests that newspaper advertisements may be more suitable than online advertisements to reach older people and to appeal them to participate in clinical studies of age-related diseases. However, the current evidence on methods that recruit web-based clinical studies are mainly based on those associated with smoking cessation [8], mental health [1], hypertension [17], or cancer [18]. Evidence was especially limited in terms of their employment in the field of neurological diseases, except for a very few earlier studies using online recruitment for prevention trials of Alzheimer disease (AD) [19].

Individuals with preclinical AD, which corresponds to the presymptomatic stage of AD where patients exhibit the earliest pathological changes in the brain but without significant cognitive decline [20-23], have now been focused on as primary targets of AD clinical trials. As they are asymptomatic, AD researchers often recruit participants via an online screening registry. This method has a problem similar to earlier web-based clinical interventions in other domains: how to promote recruitment for preclinical AD screening studies with limited research funds while incorporating the characteristics of preclinical AD individuals as the target group.

**Figure 1:**
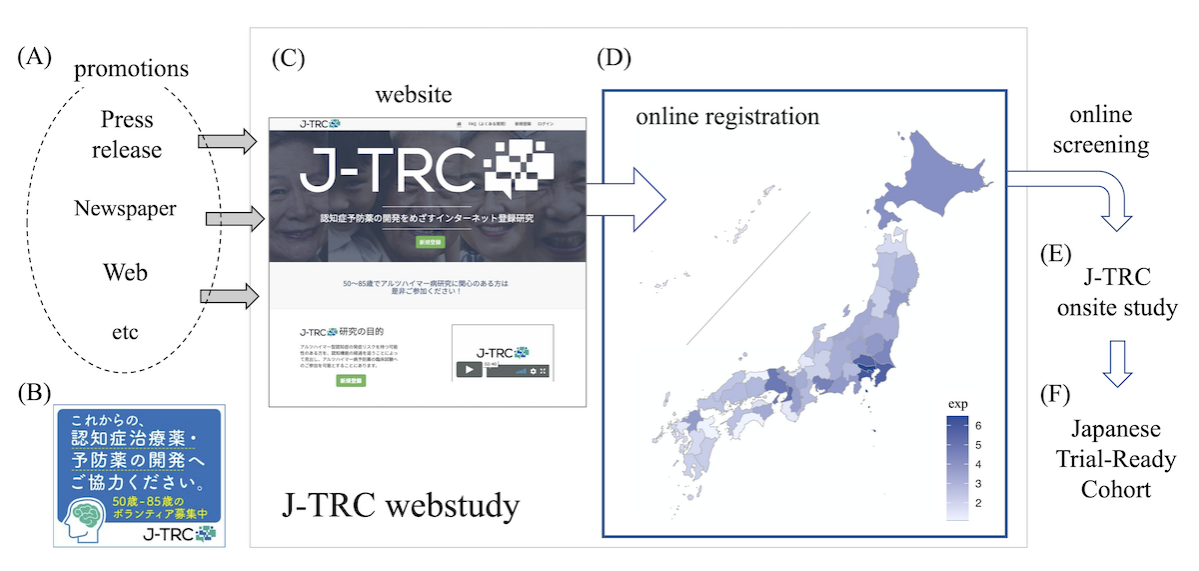
Schematic outline of the study. Multiple methods (A) including web banner advertisement (B) were used to promote visitors and registrants to J-TRC website (C, D). Among them, we will recruit individuals with higher risk of amyloid positivity to participate in the J-TRC onsite study (E, F). J-TRC: Japanese Trial-Ready Cohort.

Since 2019, we have been conducting the Japanese Trial-Ready Cohort (J-TRC) web study [24] (limited to Japanese domestic access only) (Figure 1), as one such online screening registry of preclinical AD [25]. Within the first 9 months of its 2019 launch, the J-TRC web study has recruited more than 4000 online participants from all over Japan [26] via multiple modalities of promotions (Figure 1A), including press releases, newspaper advertisements, web advertisements (Figure 1B), and direct email invitations sent to another related registry. Among the website visitors, those who were willing to participate in the study proceeded to study registration (Figure 1C) and were screened for their web-based cognitive performance every 3 months to further refer them to the successive in-person study (Figure 1E), aiming to build a large Japanese cohort of preclinical individuals ready for the AD clinical trials (Figure 1F).

Studies of the detailed features or differences in the effectiveness of these promotion methods in the context of the J-TRC web study promotion will be informative for the future planning of promotions for similar settings of presymptomatic online screening registries, regardless of the disease domain. However, in the J-TRC web study promotion, these promotion methods were conducted using a combined approach, with unignorable overlap in their timings during the same period. Thus, a direct comparison of the effectiveness between these different methods is impossible. Therefore, by applying a multivariate time-series analysis, we quantitatively evaluated the efficacy and cost-effectiveness of each of these promotion methods. Our study will also be informative to fill the gap in evidence about the difference in the effectiveness of several methods to promote web-based clinical studies between different countries (primarily between Japan and Western countries), since the earlier online clinical studies have come from Western countries where clinical trial environments are different from those in Japan.

## Methods

### Ethics

The J-TRC web study was approved by the University of Tokyo Graduate School of Medicine Institutional Ethics Committee (ID: 2019132NI-(3)), and online informed consent was obtained from each participant upon registration for the analysis during collection of basic demographic information of the registrant. Upon using the J-TRC website, users are required to agree to the terms of use and the privacy policy of the website.

### J-TRC Web Study Promotions

The details about the J-TRC web study were described in our recent report [26]. We were helped by Hakuhodo Inc [27] for the advertising and public relations of the J-TRC web study. The J-TRC web study has recruited more than 4000 online registrants from all over Japan within the first 9 months of its launch in October 2019. The promotion was conducted using multiple advertisement methods (Figure 1A), including a press release concurrent with the launch, advertisements in national newspapers, online advertisements, and direct email invitations to participants of another cognitive registry. These promotions were essentially performed one by one, but their timing interval, frequency, and duration period differed greatly, and they may have overlapped at some points. Financial incentives (eg, reward payments) [10] were not used. In this study, we reviewed the time-series data of promotions and the successive change in the target metrics during the consecutive period from October 31, 2019 to July 16, 2020 (for a total of 260 days).

After we conducted an initial press release simultaneously with the web study launch on October 31, 2019, the launch was reported by the media via newspapers or their websites or introduced by health-related web articles. This motivated target individuals to access the J-TRC web study website (we refer to this type of promotion as “press release reports” here). The J-TRC web study website is written in Japanese, and access to it is limited to domestic connection only. Since these press report activities were not advertisements and solely relied on the voluntary work of news media, we could not directly control the degree and range of the reports’ exposure in the media. Costs required for the initial press release, including the venue and equipment fee, was Japanese yen (JP ¥)7,620,000 in total. A currency exchange rate of US $1 = JP ¥108 was applicable on October 31, 2019. We confirmed the published date of newspaper reports or website articles introducing the J-TRC study using Nikkei Telecom [28], a large Japanese database covering newspapers, TV news, and magazines published in Japan. The impression data for each article were not available. Reports on radio news or other social media were not reviewed, as we were not able to access the reports on these media. The promotion press release reports were further separately treated, depending on the date of reports: whether it was reported within the first week (during which promotion activity was performed) or later, following the launch of the J-TRC web study.

Advertisements on the web were conducted in 2 ways: (1) listing advertising and (2) banner advertising. The listing advertisements (referred to as “web listing advertisements” here) used Google Ads [29] and Yahoo! Japan Ads [30] to display advertising texts recruiting J-TRC web study participants by specifically targeting internet users who were 60 years or older and searched for keywords associated with dementia, such as “物忘れ” (a Japanese word meaning “memory loss” or “memory impairment”) , “記憶障害” (ie, “memory loss” or “memory impairment”), “認知症” (ie, “dementia”) , “アルツハイマー病” (ie, “Alzheimer disease”) , “レビー小体型認知症” (ie, “dementia with Lewy Bodies”) , or “軽度認知障害” (ie, “mild cognitive impairment”) on the Google or Yahoo! search engines. Clicking the advertising sentence displayed on the search result directly connected the user to the J-TRC website (Figure 1C), which is counted as a onetime website visit. The listing advertising was conducted during the period from December 12, 2019 to January 25, 2020 (for a total of 42 days), with a total budget of JP ¥4,200,000. There were 2,919,186 impressions, among which there were 24,024 clicks (click rate 0.82%) in total with Yahoo! Japan Ads (JP ¥114 per visitor). There were 428,104 impressions, among which there were 11,183 clicks (click rate 2.61%) in total with Google Ads (JP ¥123 per visitor).

Web banner advertising promotion (referred to as “web banner advertisement” here) was conducted during the period of Dec 12, 2019 to Mar 20, 2020 (for a total of 100 days), with a total budget of JP ¥1,468,000. Web banner advertisement, as exemplified in Figure 1B, was displayed on the dementia-related web article pages of Medical Note [31], a medical web media. Clicking the banner advertising also directly transferred the user to the J-TRC website (Figure 1C), as with the web listing advertisement. This advertisement received 286,231 impressions, of which 1,138 produced clicks (click rate 0.4%) in total (JP ¥1289 per visitor), as summarized in Table 1.

**Table 1:** Summary of costs required for each promotion method.

Promotion method	Total cost, JP ¥^a,b^	Total visitors to website	Actual cost per visitor, JP ¥^b^
Press release reports (1st and 2nd week+)	7,620,000	—^c^	—
Newspaper advertisement	6,300,000	—	—
Web listing advertisement	4,102,124	35,207	116.6
Web banner advertisement	1,468,000	1,138	1289
Email invitation to IROOP^d^	0^e^	—	—

^a^JP ¥: Japanese yen.

^b^A currency exchange rate of US $1 = JP ¥108 was applicable on October 31, 2019.

^c^—: not available.

^d^IROOP: Integrated Registry of Orange Plan.

^e^Sending the emails did not require any cost by itself.

Advertisements in the national newspaper (referred to as “newspaper advertisement” here) were conducted twice in total. The first ran on February 13th, 17th, and 19th of 2020 in The Yomiuri Shimbun [32] morning and evening editions, with a total cost of JP ¥2,300,000, with advertisements announcing the holding of a public seminar on the latest dementia research and the introduction of the J-TRC study. The second promotion ran on March 21, 2020 in The Yomiuri Shimbun morning edition, with a cost of JP ¥4,000,000 to advertise the J-TRC study directly. Advertisements in local and regional newspapers were not used.

The direct email invitations were sent twice in total to participants in the Integrated Registry of Orange Plan (IROOP; referred to as “email invitation to IROOP” here) [33]. The IROOP is a multiple-layered cognitive registry aimed at developing a better understanding of dementia, improving dementia care management, and facilitating dementia clinical trials [33]. The IROOP participants receive regular cognitive tests and some useful information about dementia clinical studies. Sending the emails did not require any cost by itself.

### Effectiveness Assessment of Promotions

**Figure 2:**
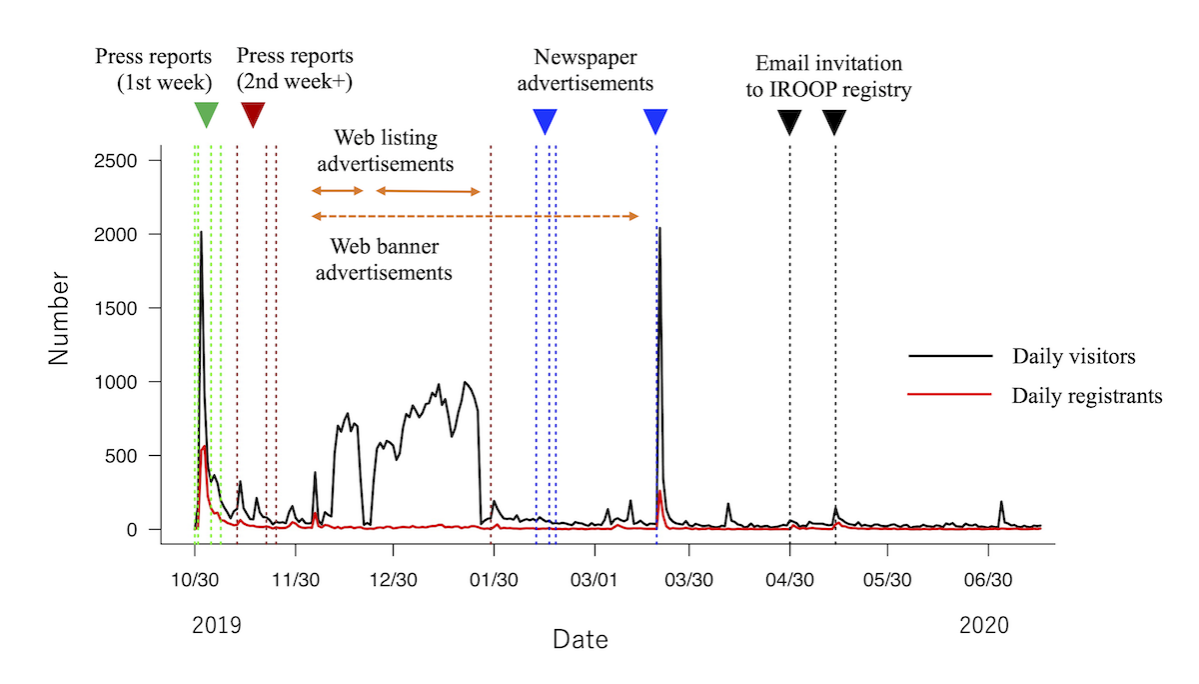
Serial daily record of the number of web study website visitors and study registrants. Exposure dates for each promotion method are represented by the vertical or horizontal lines, as labeled. IROOP: Integrated Registry of Orange Plan.

As shown in Figure 2, the timing interval, frequency, and duration of each promotion method differed greatly: these promotions were conducted in a combined manner. Figure 2 shows a time-series increase in the number of daily visitors to the J-TRC web study website (black line) and the number of daily registrants to the J-TRC web study (red line). Exposure to each promotion method is represented by the vertical or horizontal lines: green lines correspond to the day when press reports were published within the first week after the study launch, brown lines correspond to the press reports published after the first week since the launch, blue lines correspond to the day of newspaper advertisements, and the black lines correspond to the day when email invitations to the IROOP registry were sent. The orange horizontal lines correspond to the period during which web listing advertisements were carried out, and the orange horizontal dotted line corresponds to the period during which web banner advertisements had been displayed.

We cannot always clearly separate which promotion method, by itself, led to increases in the target metrics (eg, daily number of visitors or registrants in Figure 2), so we used the vector-autoregression (VAR) model as a multivariate time-series analysis to avoid cross-contamination on the effects of different promotion methods. We used R package vars [34] to perform the VAR model calculation, and the adequate lag order was determined to be 1 based on the Akaike’s Information Criteria. The following equations describe the VAR model used in this study with a lag order of 1:

 Y_1, t_ = C_1_ + φ_11_Y_1,t-1_ + φ_12_Y_2,t-1_+ φ_13_Y_3,t-1_+ φ_14_Y_4,t-1_+ φ_15_Y_5,t-1_+ φ_16_Y_6,t-1_+ φ_17_Y_7,t-1_+ φ_18_Y_8,t-1_ + ε_1,t_** (1)**

Y_k, t_ = C_k_ + φ_k1_Y_1,t-1_ + φ_k2_Y_2,t-1_+ φ_k3_Y_3,t-1_+ φ_k4_Y_4,t-1_+ φ_k5_Y_5,t-1_+ φ_k6_Y_6,t-1_+ φ_k7_Y_7,t-1_+ φ_k8_Y_8,t-1_ + ε_k, t_ (k = 2,3, ... 8)** (2)**

Value Y_1_ is one of the target metrics we aimed to evaluate, representing the number of daily new visitors to the J-TRC website (ie, “daily visitors” in this study), the number of daily new registrants to the J-TRC web study (ie, “daily registrants”), the daily registration rate among daily visitors (registration rate = daily visitors/registrants; ie, “daily conversion rate”), daily rate of eligible registrants to the J-TRC web study among all daily registrants (eligible registrant rate = eligible registrants/total registrants; ie, “daily eligibility rate”), or the median age of daily eligible registrants (ie, “registrants’ age”). Daily data of these metrics and other eligible participants’ demographics were extracted from the data server of the J-TRC web study, for which management is entrusted to Nittetsu Hitachi Systems Engineering, Inc [35]. Eligibility for participation in the J-TRC web study was defined as follows: participants who had completed the registration and demographic input, provided informed consent for study participation, had no prior history of a dementia or AD diagnosis, and were aged from 50 to 85 years old at the time of registration [26].

Values Y_2_ through Y_7_ are the daily binary exposure statuses (with or without, regardless of the degree of impressions) to each of the promotion modalities as follows: Y_2_ stands for the initial press release reports within the first week since launch, Y_3_ stands for the press release reports after the first week since launch, Y_4_ stands for the national newspaper advertisements, Y_5_ stands for the web listing advertisements, Y_6_ stands for the web banner advertisements, and Y_7_ stands for the email invitations to IROOP. In addition, we used Google Trends’ [36] results to incorporate general nationwide trends [37] of interest in dementia in Japan, irrespective of the promotional activities of the J-TRC study. We allocated Y_8_ to the daily trend in the relative search volume in Google Trends, where the maximum daily count of searches for “dementia” (in its corresponding Japanese keyword) within the included period is equal to 100%. Trends in social media such as Twitter were not incorporated, because the J-TRC web study and its related keywords had hardly ever been tweeted about (as confirmed in September 2020). As we had confirmed that a few series of variables, including Y_1_-Y_8_, were not stationary via the augmented Dickey-Fuller test with R package tseries [38], we did not calculate Granger causality. The obtained coefficients ϕ_12_ through ϕ_18_ in Equation 1 correspond to the effect sizes of change in the target metric Y_1_ at 1 day following the application of each promotion modality (Y_2_ through Y_8_) on the previous day.

The coefficients of promotion methods were further used to derive cost-effectiveness (costs in JP ¥ required to achieve 1 J-TRC website visitor or 1 J-TRC web study registrant). Although the total number of impressions and clicks following the use of advertisements were available in the case of web advertising, such data were not available for press releases, newspaper advertisements, or email invitations. Therefore, we calculated the average cost-effectiveness of each promotion method using the following equation:

Mean cost-effectiveness = (Total cost of 1 promotion method) / (Estimated coefficients of the method [for daily visitors or daily registrants] × Total days spent on that method) **(3)**

The cost-effectiveness of email invitations to the IROOP registry could not be evaluated adequately as it required no substantial costs.

### Validation

We additionally conducted promotions by newspaper advertisements alone in February to March 2021, of which data we used to validate the effectiveness of newspaper advertisements as calculated above, thereby attempting to further reduce the risk of cross-contamination of different promotion methods. This was again examined by applying the VAR model, where included variables were limited to Y_4_ and Y_8_ in Equation 1 and Equation 2.

### Statistical Analysis

All the above data handling and analyses were performed using R 3.5.1 (R Foundation for Statistical Computing, Vienna, Austria). A *P* value <.05 was considered as significant if not otherwise mentioned. To summarize the continuous value, we used the mean and SD. To summarize the geographical distribution of eligible registrations across Japan, we used R packages choroplethr [39] and choroplethrAdmin1 [40].

## Results

### Overview of Serial Daily Records in the Target Metrics

During the review period from October 31, 2019 to June 17, 2020, there were 48,334 website visitors, 4429 registrations (eligible or not; 9.16% of all 48,334 website visitors), and 3081 eligible registrations (69.56% of all 4429 registrations). The basic demographics of the eligible participants were as follows: mean age was 62.3 (SD 8.8) years at registration, 54.79% (1688/3081) were female, and 40.28% (1241/3081) had a family history of dementia or AD. Prefecture-level geographical distribution of the number of eligible registrants across Japan is shown as a choropleth map with a scale of natural logarithms in Figure 1D. Regional differences in the number of eligible registrants are apparent, reflecting the basic population by prefecture.

Time series of the number of daily visitors to the website and the number of daily registrants to the J-TRC web study are plotted in Figure 2. By visual inspection, both the number of daily new visitors (black line) and the number of daily new registrants (red line) prominently increased during the first week, along with the initial press release reports (represented by green vertical lines). The number of daily visitors gradually decreased thereafter, occasionally showing a transient mild surge following the press release reports and web article publications. Not all the small surges correspond with the timings of the press release reports and web articles we annotated (represented by green or brown vertical lines), partly because we cannot capture all the published newspaper, web, or magazine articles by Nikkei Telecom. Web advertisements (listings or banners, represented by horizontal orange lines) greatly contributed to the number of daily visitors but seemed not to affect that of daily registrants. Newspaper advertisements (represented by blue vertical lines) significantly contributed to the transient increase in daily visitors (*P*<.001) and daily registrants (*P*=.001). Email invitations to IROOP registrants seemed to mildly increase the number of daily visitors. During the period when no promotions were conducted (eg, June 2020), the mean number of daily visitors was 23.4 (SD 8.5) visitors per day, and the mean number of daily registrants was 1.6 (SD 1.5) registrants per day.

### Efficacy of Advertisements in Vector Autoregression Model

We quantitatively assessed the above visual inspection by multivariate time-series analysis using the VAR model. Table 2 presents the result coefficients and their adjusted *P* values in the VAR model with a lag order of 1. When we had employed 1 promotion method (shown in rows) over 1 day, the contribution of that method in changing the target metric (shown in columns) on the next day (with delay=1 day) is quantified as the coefficient value of the corresponding cell. For example, the press release reports at 1 day (within the first week) contributed to an increase of 716.6 in daily website visitors on the next day.

**Table 2:** Result coefficients of vector-autoregression model (2019-2020).

Promotion method			Y_1_ (effect on):								
			Daily visitors		Daily registrants		Daily conversion rate		Daily eligibility rate		Registrants' age
**Y_2_ (press release reports, 1st week)**											
	Result coefficient	716.6		183.6		8.9		–8.4		0.8	
	*P* value	<.001		<.001		.06		.54		.84	
**Y_3_ (press release reports, 2nd week+)**											
	Result coefficient	88.9		11.8		0		–7.5		–3.7	
	*P* value	.28		.52		.99		.53		.28	
**Y_4_ (newspaper advertisements)**											
	Result coefficient	509.3		64		–2.2		–1.3		–2.4	
	*P* value	<.001		.001		.60		.92		.50	
**Y_5_ (web listing advertisements)**											
	Result coefficient	486.6		6.7		–4.5		–14.8		–4.4	
	*P* value	.001		.38		.008		.003		.002	
**Y_6_ (web banner advertisements)**											
	Result coefficient	–40.4		–6.2		–2.5		2.5		2.3	
	*P* value	.12		.29		.06		.50		.03	
**Y_7_ (email invitations to IROOP^a^)**											
	Result coefficient	–3.1		23.1		43.3		8.9		2.6	
	*P* value	.98		.38		<.001		.60		.59	
**Y_8_ (trends in general interest in dementia, from Google Trends)**											
	Result coefficient	1.1		0.1		0		0.1		0	
	*P* value	.21		.52		.45		.55		.23	

^a^IROOP: Integrated Registry of Orange Plan.

Overall, on average, press releases within the first week had the highest effect on increasing daily visitors (+716.6), followed by newspaper advertisements (+509.3), and the web listing advertisements (+486.6). The press releases within the first week had the highest effect on increasing the daily registrants (+183.6), followed by newspaper advertisements (+64). In addition, email invitations to IROOP registrants showed the highest effect on increasing the daily conversion rate (+43.3%), whereas web listing advertisements showed the opposite effect, lowering the daily conversion rate (–4.5%) and the daily eligibility rate (–14.8%). Furthermore, web banner advertisements contributed to making the median age of daily eligible registrants slightly older (+2.3 years old), while the web listing advertisement contributed to lowering the median age of daily eligible registrants (–4.4 years old). General trends of interest in the keyword “dementia” by the Japanese population, as represented by the relative search volume in Google Trends, had no effect on increasing nor decreasing the daily target metrics.

### Cost-effectiveness Analysis

The average cost-effectiveness per website visitor was calculated using the VAR model coefficients (Table 2). Only promotion methods with a coefficient value higher than 0 were included in the calculation, because we did not assume a negative value in the number of visitors and registrants. The results are shown in Table 3. The results for web banner advertisements were negative and, therefore, not applicable.

**Table 3:** Cost-effectiveness analysis.

Promotion method	Total days of advertisement	Estimated visitors per 1 day of advertisement	Average cost per visitor, JP ¥^a,b^	Estimated registrants per 1 day of advertisement	Average cost per registrant, JP ¥^b^
Press release reports (1st week)	4	716.6	2658	183.6	10,376
Press release reports (2nd week+)	4	88.9^c^	21,429^c^	11.8^c^	161,440^c^
Newspaper advertisements	4	509.3	3093	64	24,609
Web listing advertisements	42	486.6	201	6.7^c^	14,592^c^
Web banner advertisements^d^	100	N/A^e^	N/A	N/A	N/A

^a^JP ¥: Japanese yen.

^b^A currency exchange rate of US $1 = JP ¥108 was applicable on October 31, 2019.

^c^The efficacy coefficient of these promotion methods were not significant (in Table 2), and, therefore, presented for reference only.

^d^The results for web banner advertisements were negative and therefore not applicable.

^e^N/A: Not applicable.

The press release reports within the first week and the newspaper advertisements had similar levels of cost-effectiveness, at approximately JP ¥3,000 per website visitor (JP ¥2,658 and JP ¥3,093, respectively; Table 3). The average cost of the web listing advertisements was calculated as JP ¥201 per visitor, which is within the same order as its direct cost-effectiveness (ie, JP ¥116.6; Table 1). In terms of the cost-effectiveness per registrant (Table 3), newspaper advertisements required the highest cost (JP ¥24,609 per registrant), followed by press release reports within the first week (JP ¥10,376 per registrant).

For reference, we also overviewed the cost-effectiveness of promotion methods, for which efficacy was not significant (*P* values shown in Table 2) in the current VAR model: the web listing advertisements had a similar level of cost-effectiveness for registrant recruitment (JP ¥14,592) as the press releases within the first week or as the newspaper advertisements. In addition, the press release reports in the second week or later had the worst level of cost-effectiveness (JP ¥21,429 per visitor and JP ¥161,440 per registrant).

**Figure 3:**
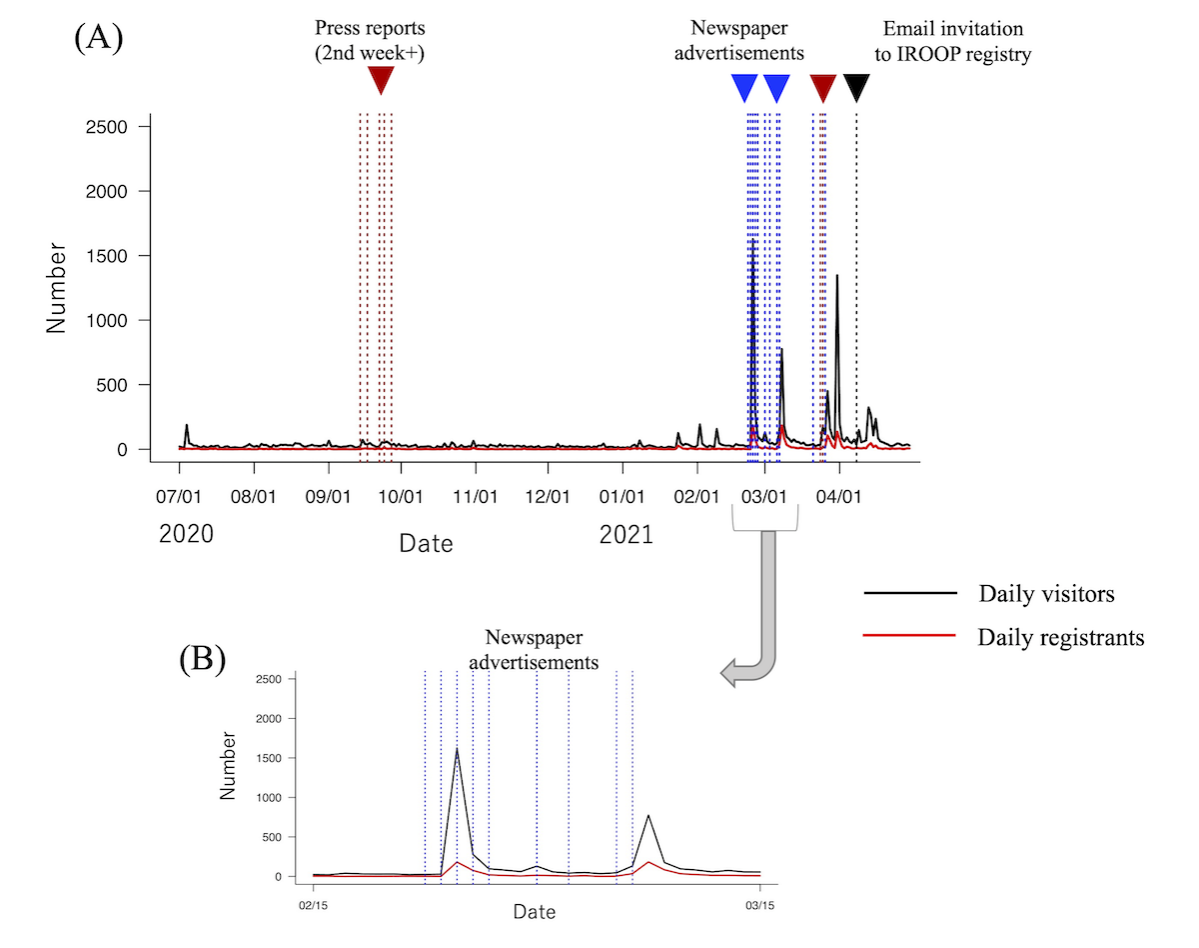
Serial daily record of the number of web study website visitors and study registrants, just succeeding the data presented in Figure 2. (A) Exposure dates for each promotion method are represented via vertical lines, as labeled. (B) More detailed exposure dates for the promotion method newspaper advertisements are represented via vertical lines. IROOP: Integrated Registry of Orange Plan.

Lastly, we validated the above results of newspaper advertisements in a period when there were no other promotions used, in 2021. Figure 3 shows the time series of the number of daily visitors to the website and the number of daily registrants to the J-TRC web study, just succeeding the data presented in Figure 2. During 2020, since April, we could not conduct any active promotions mainly due to the COVID-19 pandemic. Then, in February 2021, we decided to conduct promotions mainly by using newspaper advertisements, which we considered the most effective promotion method based on the results just described. In a period between the middle of February 2021 and the middle of March 2021, there were no other promotion methods conducted (Figure 3B), so that analyzing the serial changes in this period would allow us to see the pure effect of newspaper advertisements. The result coefficients of the VAR model are shown in Table 4. Although a bit smaller than the coefficients in Table 2, on average, newspaper advertisements showed a similar level of significant increases to the daily visitors (+295.2, *P*=.032) and daily registrants (+42.7, *P*=.025).

Calculated cost-effectiveness of newspaper advertisements, in this case, was JP ¥5226 per website visitor and JP ¥36,156 per registrant, which were only approximately 1.5× higher than the results shown in Table 3.

**Table 4:** Result coefficients of vector-autoregression model (2021).

Promotion method			Y_1_ (effect on):								
			Daily visitors		Daily registrants		Daily conversion rate		Daily eligibility rate		Registrants' age
**Y_4_ (newspaper advertisements)**											
	Result coefficient	295.2		42.67		5.42		–9.65		0.4	
	*P* value	.03		.03		.13		.35		.90	
**Y_8_ (trends in general interest in dementia, from Google Trends)**											
	Result coefficient	–1.64		–0.34		–0.1		–0.12		0.05	
	*P* value	.75		.63		.47		.77		.69	

## Discussion

### Principal Findings

In this study, using multivariate time-series analyses, we quantitatively examined the recruitment efficacy of multiple types of promotion methods and their cost-effectiveness. Our results showed that each promotion modality had different features in their effects on website visitors and participant recruitment for the J-TRC web study. Under the advertising condition settings so far, the traditional methods of press releases and newspaper advertisements had a marked effect on increasing website visitors and study registrants with fair cost-effectiveness mostly equivalent to earlier studies while web-based advertisements also had a considerable effect on increasing website visitors with highly efficient costs. In addition, direct email invitations to other cognitive registries achieved the highest registration rate, and 2 web advertising methods altered the distribution of the registrants’ age mildly and oppositely. These results can provide important suggestions for future promotion for recruiting presymptomatic participants to AD clinical trials in Japan.

Although the promotion methods press release reports after the first week, web banner advertisements, or email invitations to IROOP registry seemed to have some effects on the number of daily visitors by visual inspection (Figure 2), these effects were not statistically significant. These results are those derived in the framework of the VAR model and under the advertisement settings so far, so careful interpretation is needed, as they may actually turn out to be effective in other statistical models or in other advertisement settings. For example, prolonging the period of web banner advertisements (eg, to 1 year or more, as in earlier studies [1]), changing the placement of the banner advertisement on the website, or using banner images with more visually appealing designs [11,41] may yield different results.

Similarly, these results do not always mean that web listing advertisements are not effective at recruiting registrations to the J-TRC web study. Because many earlier online studies have repeatedly reported the efficacy of web advertisements [3-6,8,9,11-13], much needs to be done to improve the efficacy of web listing advertisements in the J-TRC web study. The web listing advertisements contributed to increasing daily visitors greatly but did not greatly affect the daily registrants, suggesting a large proportion of visitors left the website without proceeding to registration. In addition, the web listing advertisements also decreased the rate of eligible participants among the registrants (Table 2), implying that website visitors via web listing advertisements may have been less eager to participate in the J-TRC web study, because many of our noneligible registrants had not fully completed their basic demographic information. Based on these points, it may be helpful to link the web advertisement to the landing web page, which motivates website visitors to participate in the web study.

The different promotion methods seemed to have recruited participants with slightly different backgrounds. For example, direct email invitations to other cognitive registries achieved the highest registration rate, presumably reflecting their high interest in the AD clinical studies [5]. We can, therefore, expect these registrants to have high eligibility as participants for the J-TRC study as well as successive AD clinical trials, which require years of continuous commitment from participants. In addition, 2 web advertising methods had incongruent effects on recruiting participants of slightly different ages, which we consider to be due to the difference in the age of the main users. Namely, the main users of the webpage where the web banner was displayed might have been slightly older than those who searched for dementia-related keywords by search engines. These features suggest that changing the allocation of promoting methods may help adjust the background of overall J-TRC study participants in a manner optimized for the screening cohort for preclinical AD.

We calculated the average cost-effectiveness by using coefficients in the VAR model, because data of daily visitors and registrants were not directly available, except for the data tracked by the web advertisements. The direct cost-effectiveness for web listing advertisements was JP ¥117 per visitor; the calculated average cost-effectiveness for the same web listing advertisements were similar, at JP ¥201 per visitor. This result supports the reliability of the average cost-effectiveness calculated in our study, suggesting we could compare the rough cost-effectiveness between the different promotion methods.

Traditional methods of press releases and newspaper advertisements were suspected to have the largest effect on increasing website visitors and study registrants, despite the considerable costs required. This may be due to the nature of newspapers: it was reported that the newspaper was considered as a highly important source of information and as the most reliable media by Japanese people in their 60s, when compared with television or internet [16]. The cost-effectiveness of newspaper advertisements was JP ¥24,609 per registrant, which is mostly equivalent to the costs reported in earlier studies recruiting via newspaper advertisements in Western countries, such as approximately UK £250 (US $322 on October 31, 2019) per eligible participant for a celecoxib clinical trial in Britain [14], Can $113 (US $86 on October 31, 2019) per enrolled participant for recruiting postmenopausal women in Canada [42], US $115 per recruited participant for a smoking cessation intervention [43], and Aus $239 (US $164 on October 31, 2019) per recruited eligible participant for elderly patients with diabetes in Australia [44]. Because the estimated efficacy of web advertisements to increase the J-TRC web study registrants turned out not to be significant (Table 2), we cannot compare the cost-effectiveness between newspaper advertisements and the web listing advertisements with confidence. However, the newspaper advertisements may also have a similar level of cost-effectiveness as web listing advertisements, which were reported to have a good cost-effectiveness in several earlier studies [3-6,8,9,11,12]. We also confirmed that the efficacy and cost-effectiveness of newspaper advertisements were largely stable whether they were measured in combination with other variables or measured alone with different timings (Table 2; Table 4). These points suggest that newspaper advertisements might be a reasonable method to promote registrations [13], even in the context of the J-TRC web study. The press release reports within the first week may also be effective to a certain extent, although we consider the generalizability of the effect of the press release reports to be rather limited because it cannot be conducted in replicate, unlike newspaper advertisements, nor can its degree of media coverage be controlled by researchers.

### Limitations and Future Directions

Our study had several limitations. First, the VAR model presumes that the effect of each variable is fixed throughout the included period, but this is not always guaranteed: web advertisements or newspaper advertisements that were repeatedly displayed will inevitably lessen the recruitment effect. We also regarded all different media reports as a single category of press release reports and regarded the 2 different newspaper advertisement times (the first one for event announcement and the second one for the direct J-TRC promotion) as a single category of newspaper advertisements, which could lead to an inaccurate estimation in their average cost-effectiveness. In addition, the current VAR model–based approach to estimate the number of visitors and registrants may have underestimated the true number of visitors and registrants, because the estimated efficacy of multiple promotions (as calculated by the sum of VAR significant coefficients × the total number of days of each promotion) was 25,340 visitors (among 48,334 true visitors) and 990 eligible registrants (among 3081 true eligible registrants). Although there is a similarity in the order of digits, this degree of underestimation in the efficacy of valid promotion methods suggests the true cost-effectiveness of the press releases and newspaper advertisements may be 2-3× lower than the estimated one reported here. This should be taken into consideration when planning future promotion strategies.

For complementing the shortness of this analysis, it may be helpful to follow up by determining the specific J-TRC web study promotions viewed by each participant. For example, a future study should make an online survey for website visitors or registrants asking how they learned of or were referred to the J-TRC study. However, it may not be easy to carry out within the current system, which was adopted from the Alzheimer Prevention Trials web study used in a similar preclinical AD study in the United States [25]. To use a data tracking system when sending emails to participants of other cognitive registries may be easier to implement.

In the future, we must address the effectiveness of social media such as Facebook, Twitter, Instagram, LINE, and TikTok to recruit elderly participants in the context of a Japanese clinical trial environment. Outside Japan, Facebook has been shown to be effective to recruit elderly participants to clinical trials [17,45]. In Japan, although the use rate of Facebook by elderly people has been relatively lower compared with younger people, it has been increasing in recent years. Facebook, Twitter, and Instagram were reported to have similar use rates, of approximately 10% in 2019 [16]; therefore, we can expect promotion via Facebook, Twitter, or Instagram might also be increasing in feasibility in Japan. Moreover, the use rate of elderly users of LINE, an instant communication app, dramatically increased between 2012 and 2019, and LINE is now the most popular social media platform in Japan (with approximately 70% use rate among people in their 60s in 2019) [16], meaning LINE may be one of the most useful media for promoting recruitment for the J-TRC web study, such as via web banner advertisements.

### Conclusions

We quantitatively evaluated the efficacy and cost-effectiveness of multiple methods to promote the J-TRC web study. Our results showed that each promotion modality had different degrees of efficacy for recruiting visitors and participants to the web study website: under the advertisement setting conditions, traditional methods of initial press releases and newspaper advertisements were considered to have the largest efficacy in recruiting study registrants, with fair cost-effectiveness that was equivalent to earlier online studies of other disease domains, while web advertisements only contributed to increasing website visitors. These results can provide important suggestions for future promotion for recruiting presymptomatic participants to clinical trials of AD in Japan.

## Abbreviations

AD: Alzheimer disease
IROOP: Integrated Registry of Orange Plan
JP ¥: Japanese yen
J-TRC: Japanese Trial-Ready Cohort
VAR: vector-autoregression
